
JOURNAL INFORMATION
==============================
NLM Title Abbreviation: ScientificWorldJournal
Journal ID: ScientificWorldJournal
NLM Journal ID: 101131163
Journal ID: TSWJ

The Scientific World Journal

ISSN: 2356-6140
EISSN: 1537-744X
Publisher: Wiley

ARTICLE INFORMATION
==============================
PMCID: PMC5823158
PMID: 19219378
DOI: 10.1100/tsw.2009.11
Article ID: 750237
Article version: 1
Subjects: Directions in Science

The N-Terminal Domain of Thrombospondin-1: a Key for the Dual Effect of TSP-1 in Angiogenesis and Cancer Progression?

Morandi Verônica
Universidade do Estado do Rio de Janeiro (UERJ), Departamento de Biologia Celular, Laboratóriode Biologia da Célula Endotelial e da Angiogêse, Maracanã, Rio de Janeiro - RJ, Brazil
*Verônica Morandi: veronica@uerj.brAcademic Editor: Martin Goette

Publication date: 2009
Electronic publication date: 2009 Feb 15
Volume: 9
First page: 133
Last page: 136
Received 2008 Sep 5; Revised 2008 Dec 22; Accepted 2009 Jan 14
Copyright: Copyright © 2009 Verônica Morandi.
Copyright year: 2009
License: This is an open access article distributed under the Creative Commons Attribution License, which permits unrestricted use, distribution, and reproduction in any medium, provided the original work is properly cited.
License URL: https://creativecommons.org/licenses/by/3.0/

Keywords: thrombospondin-1, angiogenesis, heparin-binding domain, syndecan-4

==============================
Directions in Science
*TheScientificWorldJOURNAL* (2009) 9, 133–136
ISSN 1537-744X; DOI 10.1100/tsw.2009.11

TheScientificWorldJOURNAL
www.thescientificworld.com

### The N-Terminal Domain of Thrombospondin-1: a Key for the Dual Effect of TSP-1 in Angiogenesis and Cancer Progression?

Verônica Morandi

*Universidade do Estado do Rio de Janeiro (UERJ), Departamento de Biologia Celular, Laboratório de Biologia da Célula Endotelial e da Angiogênese, Rua São Francisco Xavier, 524 – PHLC sala 205, Maracanã – Rio de Janeiro – RJ, Brazil*

**E-mail**: veronica@uerj.br

*Received September 5, 2008; Revised December 22, 2008; Accepted January 14, 2009; Published February 15, 2009*

---

**KEYWORDS**: thrombospondin-1, angiogenesis, heparin-binding domain, syndecan-4

---

Thrombospondin-1 (TSP-1), the most studied of a family of five extracellular glycoproteins[1], which is expressed by cells in tissues, was first identified as a major constituent of platelet α-granules[2]. It has been implicated in essential steps of cancer progression and angiogenesis[3]. TSP-1 was identified as the first naturally occurring angiogenesis inhibitor, an activity that was initially attributed to its 140-kDa proteolytic fragment[4], and then later located to type I repeats (TSRs) and to another region known as the procollagen domain[5]. TSRs act through the CD36 receptor on microvascular endothelial cells by inducing their apoptosis[6,7]. Oncogenes suppress TSP-1 expression in many tumors, whereas tumor suppressor genes lead to increased secretion of the protein[8]. Experimental overexpression of TSP-1 in many *in vivo* models and also the naturally occurring high expression of TSP-1 in many cancer patients have been associated with a significant increase in survival rates[8]. Besides the ability to inhibit angiogenesis, the antitumoral effects of TSRs seem also to be related to the activation-latent TGF-β[9], which may in turn lead to the inhibition of tumor growth[2]. However, other recent and even seminal reports show that TSP-1 can also favor tumor progression[10].

In fact, TSP-1 stimulates the adhesion, migration, and invasion of tumor cells[10,11,12]. In certain sets of breast tumors, invasive breast cancer and stromal cells within the tumor express more TSP-1 as compared to benign lesions and normal breast tissues[13,14]. Circulating levels of TSP-1 appear to be a marker of aggressiveness in some patients, with a positive correlation with microvessel density in tumor tissues, thus suggesting a proangiogenic effect for this protein in advanced breast cancer[15]. TSP-1 associated to the surface of breast cancer cells stabilizes tumor microaggregates and mediates tumor adhesion to endothelial monolayers[16]. Venous invasion was correlated with hepatocellular carcinoma progression and low overall survival rates in patients expressing high levels of TSP-1 in their tumors[17]. In colorectal cancer, the role of TSP-1 seems to depend on tumor stage, whereas in patients bearing primary tumors, high levels of TSP-1 correlate with higher survival rates, as compared with the group expressing low TSP-1 levels[18,19]. The high expression of TSP-1 in patients with colorectal liver metastasis leads to poor prognosis[20], suggesting that the protective role conferred by inhibition of angiogenesis is overcome when cancer cells spread beyond their primary niche. Thus, additional studies

©2009 with author.
Published by TheScientificWorld; www.thescientificworld.com are clearly needed to account for the observations that TSP-1 may be inefficient or even favor malignant disease, under certain conditions.

New structural and functional clues have risen from recent studies concerning the N-terminal heparin-binding domain of TSP-1, or HBD[21]. This domain was first described as bearing the high-affinity motifs for binding heparin, sulfatides, and glycosaminoglycans. Surprisingly, the HBD has been reported as proangiogenic[22,23,24], by acting through various endothelial cell receptors, including $\alpha 3\beta 1$, $\alpha 4\beta 1$, and $\alpha 9\beta 1$ integrins; cell surface–expressed calreticulin; and, as more recently reported by our group, the cell surface heparin sulfate proteoglycan, syndecan-4[25].

We have previously found that an immobilized 18-kDa recombinant form of HBD induced tubulogenesis by human endothelial cells from umbilical veins (HUVECs), whereas intact TSP-1 induced cell quiescence, with no detectable traits of apoptosis[24]. The soluble form of the fragment failed to exert the same effect. In our recent paper[25], we showed that this effect was abrogated by an antibody against the ectodomain of syndecan-4. We found that two heparin-binding sequences, namely TSP Hep I and TSP Hep II, previously identified by Murphy-Ullrich et al. as focal adhesion–disrupting motifs[26], also blocked the angiogenic effect of the TSP18 fragment, when tested as synthetic peptides. These motifs compete with the fibronectin heparin-binding motif II (FN Hep II), the site recognized by syndecan-4 in focal adhesions[27]. TSP18 and its angiogenic-derived peptides were also able to activate the cell survival Akt/PKB pathway, a response that was dependent on the upstream activation of PKC. Inhibition of PKC activation in spread cells adhering on TSP18, TSP Hep I, and TSP Hep II by selective inhibitors of PKC led to rapid cell rounding. These observations reinforce a role for TSP-1 as an inducer of the *intermediate state of adhesion*, an adhesive status through which cells adjust their plasticity in order to grow and/or migrate, or undergo morphological differentiation, without risk of entering into a death program triggered by cell detachment (*anoikis)*. In support for such a pivotal role of HBD, Murphy-Ullrich's group[28] showed that the TSP Hep I motif also acts via CRT-LRP1 receptors (calreticulin/lipoprotein receptor-related protein 1) to induce resistance to *anoikis* in mouse embryonic fibroblasts through the PI3K/Akt survival axis.

Although the physiological relevance of the proangiogenic activity of the HBD of TSP-1 remains to be established conclusively, its role in vascular homeostasis and tissue remodeling seems more than conceivable today. The HBD is proangiogenic in different experimental models *in vivo*. TSP18 induced angiogenesis when incorporated into a matrigel pellet implanted subcutaneously in nude mice (our unpublished data). This was also observed when a recombinant 28-kDa HBD was tested in the chicken chorioallantoid membrane model[22], or when a 25-kDa native form of HBD was tested in the rabbit cornea assay[23]. Additionally, the HBD can be rapidly cleaved by several relevant proteases present in vascular, inflammatory, and tumor microenvironments[29,30]. The HBD fragments with molecular weights ranging from 16 to 50 kDa are found in the supernatant of thrombin-activated platelets, and also detected in the conditioned media of endothelial cells in culture[29]. Moreover, it was shown that a 36-kDa HBD resulting from the cleavage of TSP-1 by ADAMST1 (a protease involved in injury repair) remains bound to the endothelial matrix[31]. Resistance to the antiangiogenic effect of TSP-1 was associated to the selection of angiogenic tumor phenotypes expressing high levels of VEGF and other angiogenic inducers, which would overcome the inhibitory effects of TSP-1[32,33]. Cancer and stromal-derived proteases, known to prevail in tumoral microenvironments, could also contribute to the resistance by the production of important amounts of the proangiogenic HBD of TSP-1.

Regardless of the difficulty to organize this puzzle, the characterization of the TSP-1 receptors and their recognition motifs has been giving rise to new therapeutic tools. In both animal and human therapeutic strategies, TSR mimetic peptides (ABT-510 and ABT-526) are emerging as promising tools for future antitumoral strategies since they slow tumor growth by inhibiting angiogenesis[34]. Specific antagonists for $\alpha 3\beta 1$ and $\alpha 4\beta 1$ integrins have been developed and also represent a potential as angiogenesis-blocking drugs[3]. The identification of syndecan-4 as a new receptor mediating the proangiogenic effects of TSP-1 also brings new perspectives since mice lacking syndecan-4 exhibit significant impaired wound healing and angiogenesis[35]. Since it was shown that syndecan-4–deficient fibroblasts isolated from these animals exhibit impaired migration, it is possible that this phenotype also implicates a role for syndecan-4 null endothelial cells and their altered responses to HBD fragments generated *in situ*. A possible cooperation between syndecan-4 and the β1-integrins identified as receptors for HDB remains an open issue today.

### REFERENCES

1. Calzada, M.J. and Roberts, D.D. (2005) Novel integrin antagonists derived from thrombospondins. *Curr. Pharm. Des*. **11**, 849–866.
2. Baezinger, N.L., Brodie, G.N., and Majerus, P.W. (1971) A thrombin-sensitive protein of human platelet membranes. *Proc. Natl. Acad. Sci. U. S. A.* **68**, 240–243.
3. Kazerounian, S., Yee, K.O., and Lawler, J. (2008) Thrombospondin in cancer. *Cell. Mol. Life Sci*. **75**, 700–712.
4. Good, D.J., Polverini, P.J., Rastinejad, F., Le Beau, M.M., Lemons, R.S., Frazier, W.A., Bouck, N.P. (1990) A tumor suppressor-dependent inhibitor of angiogenesis is immunologically and functionally indistinguishable from a fragment of thrombospondin. *Proc. Natl. Acad. Sci. U. S. A.* **87**, 6624–6628.
5. Tolsma, S.S., Volpert, O.V., Good, D.J., Frazier, W.A., Polverini, P.J., and Bouck, N. (1993) Peptides derived from two separate domains of the matrix protein thrombospondin-1 have antiangiogenic activity. *J. Cell Biol*. **122**, 497–511.
6. Guo, N., Krutsch, H.C., Inman, J.K., and Roberts, D.D. (1997) Thrombospondin-1 and type I repeat peptides of thrombospondin-1 specifically induce apoptosis of endothelial cells. *Cancer Res.* **57**, 1735–1742.
7. Jiménez, B., Volpert, O., Crawford, E., Febbraio, M., Silverstein, R., and Bouck, N. (2000) Signals leading to apoptosis-dependent inhibition of neovascularization by thrombospondin-1. *Nat. Med.* **6**, 41–48.
8. Ren, B., Yee, K.O., Lawler, J., and Khosravi-Far, R. (2006) Regulation of tumor angiogenesis by thrombospondin-1. *Biochem. Biophys. Acta* **1765**, 178–188.
9. Murphy-Ullrich, J.E. and Poczatek, M. (2000) Activation of latent TGF-beta by thrombospondin-1: mechanisms and physiology. *Cytokine Growth Factor Rev.* **11**, 59–69.
10. Tuszynski, G.P., Gasic, T.B., Rothman, V.L., Nudsen, K.A., and Gasic, G.J. (1987) Thrombospondin, a potentiator of tumor cell metastasis. *Cancer Res.* **47**, 4130–4133.
11. Taraboletti, G., Roberts, D.D., and Liotta, L.A. (1987) Thrombospondin-induced tumor cell migration: haptotaxis and chemotaxis are mediated by different molecular domains. *J. Biol. Chem.* **105**, 2409–2415.
12. Wang, T.N., Qian, X., Granick, M.S., Solomon, M.P., Rothman, V.L., Berger, D.H., and Tuszynski, G.P. (1996) Thrombospondin-1 (TSP-1) promotes the invasive properties of human breast cancer. *J. Surg. Res.* **63**, 39–43.
13. Brown, L.F., Guidi, A.J., Schnitt, S.J., Van de Water, L., Iruela-Arispe, M.L., Yeo, T.K., Tognazzi, K., and Dvorak, H.F. (1999) Vascular stroma formation in carcinoma in situ, invasive carcinoma, and metastatic carcinoma of the breast. *Clin. Cancer Res.* **5**, 1041–1056.
14. Bertin, N., Clezardin, P., Kubiak, R. and Frappart, L**.** (1997) Thrombospondin-1 and -2 messenger RNA expression in normal, benign, and neoplastic human breast tissues: correlation with prognostic factors, tumor angiogenesis, and fibroblastic desmoplasia. *Cancer Res*. **57**, 396–399.
15. Byrne, G.J., Hayden, K.E., McDowell, G., Lang, H., Kirwan, C.C., Tetlow, L., Kumar, S., and Bundred, N.J. (2007) Angiogenic characteristics of circulating and tumoral thrombospondin-1 in breast cancer. *Int. J. Oncol.* **31**, 1127–1132.
16. Incardona, F., Lewalle, J.M., Morandi, V., Lambert, S., Legrand, Y., Foidart, J.M., and Legrand, C. (1995) Thrombospondin modulates human breast adenocarcinoma cell adhesion to human vascular endothelial cells. *Cancer Res.* **55**, 166–173.
17. Poon, R.T., Chung, K.K., Cheung, S.T., Lau, C.P., Tong, S.W., Leung, K.L., Yu, W.C., Tuszynski, G., and Fan, S.T. (2004) Clinical significance of thrombospondin expression in hepatocellular carcinoma. *Clin. Cancer Res.* **10**, 4150–4157.
18. Maeda, K., Nishiguchi, Y., Yashiro, M., Yamada, S., Onoda, N., Sawada, T., Kang, S.M., and Hirakawa, K. (2000) Expression of vascular endothelial factor and thrombospondin-1 in colorectal carcinoma. *Int. J. Mol. Med.* **5**, 373–378.
19. Maeda, K., Nishiguchi, Y., Kang, S.M., Yashiro, M., Onoda, N., Sawada, T., Ishikawa, T., and Hirakawa, K. (2001) Expression of thrombospondin-1 inversely correlated with tumor vascularity and hematogenous metastasis in colon cancer. *Oncol. Rep*. **8**, 763–766.
20. Sutton, C.D., O'Byrne, K., Goddard, J.C., Marshall, L., Jones, L., Garcea, G., Dennison, A.R., Poston, G., Lloyd, D.M., and Berry, D.P. (2005) Expression of thrombospondin-1 in resected colorectal liver metastases predicts poor prognosis. *Clin. Cancer Res*. **11**, 6567–6573.
21. Elzie, C.A. and Murphy-Ullrich, J.E. (2004) The N-terminus of thrombospondin: the domain stands apart. *Int. J. Biochem. Cell Biol.* **36**, 1090–1101.
22. Chandrasekaran, L., Chao-Zhen, H., Al-Barazi, H., Krutzsch, H.C., Iruela-Arispe, M.L., and Roberts, D.D. (2000) Cell contact-dependent activation of alpha3beta1 integrin modulates endothelial cell responses to thrombospondin-1. *Mol. Biol. Cell* **11**, 2885-2890.

23. Taraboletti, G., Morbidelli, L., Donnini, S. Parenti, A., Granger, H., Giavazzi, R., and Ziche, M. (2000) The heparin binding 25 kDa fragment of thrombospondin-1 promotes angiogenesis and modulates gelatinase and TIMP-2 production in endothelial cells. *FASEB J.* **14**, 1674–1676.
24. Ferrari-do-Outeiro-Bernstein, M.A., Nunes, S.S., Andrade, A.C.M., Alves, T.R., Legrand, C., and Morandi, V. (2002) A recombinant $NH_2$-terminal heparin-binding domain of the adhesive glycoprotein, thrombospondin-1, promotes endothelial tube formation and cell survival: a possible role for syndecan-4 proteoglycan. *Matrix Biol.* **21**, 311–324.
25. Nunes, S.S., Ferrari do Outeiro-Bernstein, M., Juliano, L., Nader, H.B., Woods, A., Legrand, C., and Morandi, V. (2008) Syndecan-4 contributes to endothelial tubulogenesis and survival through interactions with two motifs inside the pro-angiogenic N-terminal domain of thrombospondin-1. *J. Cell. Physiol.* **214**, 828–837.
26. Murphy-Ullrich, J.E., Gurusiddappa, S., Frazier, W.A., and Höök, M. (1993) Heparin-binding peptides from thrombospondins 1 and 2 contain focal adhesion-labilizing activity. *J. Biol. Chem*. **268**, 26784–26789.
27. Woods, A., McCarthy, J.B., Furcht, L.T., and Couchman, J.R. (1993) A synthetic peptide from the COOH-terminal heparin-binding domain of fibronectin promotes focal adhesion formation. *Mol. Biol. Cell* **4**, 605–613.
28. Pallero, M.A., Elzie, C.A., Chen, J., Mosher, D.F., and Murphy-Ullrich, J.E. (2008) Thrombospondin-1 binding to calreticulin-LRP1 signals resistance to anoikis. *FASEB J.* **22**, 1–12.
29. Morandi, V., Edelman, L., Legrand, Y., and Legrand, C. (1994) Characterization of a novel monoclonal antibody (V58A4) raised against a recombinant $NH_2$-terminal heparin-binding fragment of human endothelial thrombospondin. *FEBS Lett.* **346**, 156–160.
30. Bonnefoy, A. and Legrand, C. (2000) Proteolysis of subendothelial adhesive glycoproteins (fibronectin, thrombospondin, and von Willebrand factor) by plasmin, leukocyte cathepsin G, and elastase. *Thromb. Res.* **98**, 323–332.
31. Lee, N.V., Sato, M., Annis, D.S., Loo, J.A., Wu, L., Mosher, D.F., and Iruela-Arispe, M.L. (2006) ADAMTS1 mediates the release of antiangiogenic polypeptides from TSP1 and 2. *EMBO J*. **25**, 5270–5283.
32. Filleur, S., Volpert, O.V., Degeorges, A., Voland, C., Reiher, F., Clézardin, P., Bouck, N., and Cabon, F (2001) In vivo mechanisms by which tumors producing thrombospondin-1 bypass its inhibitory effects. *Genes Dev*. **15**, 1373–1382.
33. Fontana, A., Filleur, S., Guglielmi, J., Frappart, L., Bruno-Bossio, G., Boissier, S., Cabon, F., and Clézardin, P (2005) Human breast tumors override the antiangiogenic effect of stromal thrombospondin-1 in vivo. *Int. J. Cancer.* **116**, 686–691.
34. Zhang, X. and Lawler, J. (2007) Thrombospondin-based antiangiogenic therapy. *Microvasc. Res*. **74**, 90–99.
35. Echtermeyer, F., Streit, M., Wilcox-Aldeman, S., Saoncella, S., Denhez, F., Detmar, M., and Goetinck, P.F. (2001) Delayed wound repair and impaired angiogenesis in mice lacking syndecan-4. *J. Clin. Invest.* **107**, R9–R14.

**This article should be cited as follows:**

Morandi, V. (2009) The N-terminal domain of thrombospondin-1: a key for the dual effect of TSP-1 in angiogenesis and cancer progression? *TheScientificWorldJOURNAL* **9**, 133–136. DOI 10.1100/tsw.2009.11.
